# Mechanisms of Brain Aging Regulation by Insulin: Implications for Neurodegeneration in Late-Onset Alzheimer's Disease

**DOI:** 10.5402/2011/306905

**Published:** 2011-05-26

**Authors:** Artur F. Schuh, Carlos M. Rieder, Liara Rizzi, Márcia Chaves, Matheus Roriz-Cruz

**Affiliations:** Division of Geriatric Neurology, Department of Neurology, Clinicas Hospital (HCPA), Federal University of Rio Grande do Sul (UFRGS), Ramiro Barcelos Street 2.350, 90035-903 Porto Alegre, RS, Brazil

## Abstract

Insulin and IGF seem to be important players in modulating brain aging. Neurons share more similarities with islet cells than any other human cell type. Insulin and insulin receptors are diffusely found in the brain, especially so in the hippocampus. Caloric restriction decreases insulin resistance, and it is the only proven mechanism to expand lifespan. Conversely, insulin resistance increases with age, obesity, and sedentarism, all of which have been shown to be risk factors for late-onset Alzheimer's disease (AD). Hyperphagia and obesity potentiate the production of oxidative reactive species (ROS), and chronic hyperglycemia accelerates the formation of advanced glucose end products (AGEs) in (pre)diabetes—both mechanisms favoring a neurodegenerative milieu. Prolonged high cerebral insulin concentrations cause microvascular endothelium proliferation, chronic hypoperfusion, and energy deficit, triggering **β**-amyloid oligomerization and tau hyperphosphorylation. Insulin-degrading enzyme (IDE) seems to be the main mechanism in clearing **β**-amyloid from the brain. Hyperinsulinemic states may deviate IDE utilization towards insulin processing, decreasing **β**-amyloid degradation.

## 1. Introduction

Aging can be defined as a process that invariably causes a decline in the abilities of the individuals to face environmental stressors, leading to a dysfunction in homeostasis and an increased incidence of chronic degenerative diseases [1]. Cognitive decline, which is an important aspect of aging, is a leading cause of morbidity and mortality among the elderly, since it greatly impairs their activities of daily living and quality of life [2].

Some patterns of neurodegeneration involved in cognitive impairment are highly preserved phylogenetically in mammals—such as reduced synaptic activity in neurons, alterations in glial metabolism, and accumulation of specific metabolic products [3]. 

In this chapter we will review the relationships between some disorders of metabolism related to insulin dysfunction and cognitive decline and the importance of these alterations to the neurodegenerative process in aging-related disorders, particularly Alzheimer's disease.

## 2. Interactions between Aging and Insulin Regulation

The ultimate cause of senescence remains unknown. However, Some specific mechanisms possess major roles in regulating the aging process [4, 5]. Presently, reconciliation between the two main theories of aging would be the proposal that the cumulative effects of the Reactive Oxygen Species (ROS) and Advanced Glycation End products (AGE) leads to aging. In another words, aging would be the consequence of a biological process equivalent to combustion, that is, the burning of calories by inhaled oxygen.

In fact, dietary restriction is the only well-established mechanism proved to expand life span, as demonstrated in animal models [6, 7]. This could be related to a lower mitochondrial respiratory rate, which determines lower production of ROS and, as a possible consequence, longer life span in animals. These compounds, which originated from cellular respiratory process, could damage DNA, proteins, and structural lipids. Human body is constantly generating these molecules as natural products from cell function. To counteract the deleterious effects of ROS, there are a great number of enzymatic mechanisms that scavenge these compounds—the antioxidants. It is speculated that, with aging, there would be a dysfunction in mitochondrial activity, leading to an increased production of ROS, accompanied by a decrease in the action of the antioxidants. Through this mechanism, there would be an accumulation of damaged cellular structures and, finally, cell death [8].

The other important mechanism hypothesized to have a role in aging is the accumulation of AGEs. This process takes place when proteins are exposed to chronic elevated high levels of glucose. A nonenzymatic reaction occurs and these compounds accumulate in the body, leading to toxic effects. In fact, there are an emerging amount of evidence pointing to the link between this process with normal aging and dementia (particularly Alzheimer's disease), even in the absence of diabetes [9, 10].

## 3. Insulin and the Brain

Insulin is the most important body response to storage energy after a meal. It is a peptide hormone secreted by *β* cells in pancreatic islets, and its serum concentration increases in a direct proportion to the glucose concentration. Other substrates, like amino acids and ketone bodies, can also stimulate its secretion. Moreover, insulin secretion is under neuronal control, and the anticipation of a meal can activate the parasympathetic pathway and stimulate insulin secretion without the presence of glucose. Insulin facilitated tissue uptake of glucose from blood to immediate oxidation or to storage. Adiposity is another factor that influences insulin secretion and the expression of peripheral insulin receptors [11]. 

For a long time, the brain was thought to be an insulin-independent organ [12]. However, insulin receptors are expressed throughout the brain in neurons and glial cells, suggesting a role for this hormone in cerebral function [13–15]. The concentration of these receptors is higher in particular areas, like hypothalamus and hippocampus, where it seems to act controlling feeding and body weight [16–18]. Moreover, neurons and pancreatic *β* cells share some similatities, and it is believed that they have evolved from a common ancestral neuron that produced insulin [19]. Insulin can reach central nervous system through cincumventricular areas without blood-brain barrier (BBB) and by the action of a specific insulin receptor that transports insulin into the brain through the BBB [20, 21]. This transport mechanism has a limited action, being saturated with high substrate concentration. There is evidence that high insulin levels in bloodstream results in hyperinsulinemia in cerebrospinal fluid [22]. However, more recently, data suggest that chronic hyperinsulinemia reduced its blood-brain transport [23].

Insulin is vital for proper brain tissue function and it seems to have a general neurotrophic effect. In fact, there is a large body of evidence that this hormone has an important role as a neurotrophic factor in neurite outgrowth. As a possible consequence of this primary mechanism, insulin can influence synaptic plasticity, learning, and memory processes. Insulin also regulates the internalization of neurotransmitters at several receptor sites [24].

The signal transduction after the binding of insulin in its surface receptor (insulin receptor—Irc) is complex, with the activation of a great amount of second messengers. The Irc receptor, when activated by insulin, has an intrinsic tyrosine kinase activity, forming phosphotyrosine residues that are dock sites to adaptor proteins, like insulin receptor substrates 1 and 2 (IRS1, IRS2). These molecules activate other proteins, initiating several signaling cascades. One of them is the lipid kinase phosphatidylinositol3-kinase (PIK3), which is responsible for almost all of the metabolic actions of insulin. PIK3 acts in the membrane phospholipid PIP2 (phosphatidylinositol 4,5 biphosphate) turning it into PIP3 (phosphatidylinositol 3,4,5 triphosphate). PIP3 recruits PKB (protein kinase B, also called Akt), which targets GSK3 (glycogen synthase kinase 3). Phosphorylation of GSK3 by PKB causes its inactivation, reducing the phosphorylation of glycogen synthase (GS). This decreased phosphorylation leads to a more active GS metabolite, which increases the conversion of glucose-6-phosphate to glycogen. Besides this actions related to glucose metabolism, insulin receptor activation also acts through the MAP kinase pathway affecting general gene expression [25].

Insulin also participates in many other complex reactions in the brain through different mechanisms which are less intrinsically related to aging and, therefore, stands outside the scope of this review (see [24] for a review).

## 4. Metabolic Disorders and Cognitive Decline

**Figure 1:**
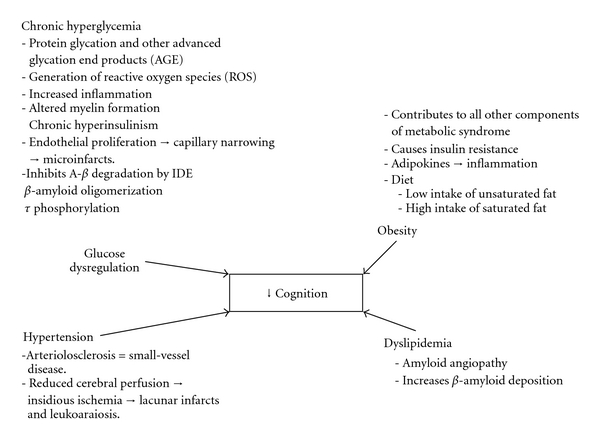
Key components of the Metabolic Syndrome (Met.S), and their possible mechanisms leading to neurodegeneration and cognitive decline. All Met.S components may contribute to cause cerebral small-vessel disease, neurodegeneration, and cognitive decline. However, obesity-related insulin resistance, causing hyperinsulinism, is thought to be the unifying pathophysiological mechanism for the development of Met.S. Aging is associated with increased insulin resistance and Met.S prevalence, and may also aggravate the severity/control of most components of this syndrome. Adapted, with authorization, from Roriz-Filho et al. 2009 [27].

In the last decade there has been a rapid increase in publications focused on the relationships between metabolic disorders, such as diabetes, obesity and metabolic syndrome, and a higher risk for cognitive decline and dementia (Figure 1). The finding that metabolic disorders may increase the risk of dementia has raised many new questions, whose answers may have critical implications, such as the way we define and classify the dementias [26].

The peripheral insulin resistance syndrome occurs when tissues become less responsive to the effects of insulin, affecting its actions on cellular glucose uptake and regulation of blood glucose levels. It is typically accompanied by compensatory hyperinsulinemia in the periphery, which has independent toxic deleterious effects upon cells. Insulin resistance is thought to be the underlying cause of metabolic syndrome and type-2 diabetes (DM.2), having, in many cases, an important role also in the development of vascular disorders such as hypertension and cardiovascular disease [28]. 

DM.2 can be considered as a pole of the insulin resistance syndrome spectrum and, just like dementia, is also a common age-related chronic disorder. About 250 million people around the world suffer from this disease, and 6 million new cases are reported each year [29]. Its prevalence increases with age: from 12% in people aged 65 to 70 years to 15% in people over age 80 [30]. This will represent an even greater burden in the near future, since the population older than 60 is expected to increase by 50% in the next 20 years (*The US Census Bureau*). Diabetes is a multisystemic disorder which could damage any organ in the body. Pathologic changes can occur in both large and small vessels, cranial and peripheral nerves, skin, and eyes. These changes have been traditionally associated with renal failure, vision loss, autonomic and peripheral neuropathy, peripheral vascular disease, myocardial infarction, and cerebrovascular disease [31].

The associations between diabetes and stroke, as well as those between diabetes and vascular dementia (VD), are already well established, being of obvious association [32–34]. Nevertheless, the connections between diabetes and other forms of cognitive decline seem to involve more subtle and complex mechanisms.

In the last years, the line border separating Alzheimer Disease (AD) from VD has become blurred [35]. VD is a heterogeneous disorder which can have its pathological presentation ranging from multiple macroinfarcts to small-vessel ischemic disease or microvascular injury. Biswanger disease is a subtype of vascular dementia caused by several brain microinfarcts and/or a by a chronic oligaemic state leading to white matter degeneration [36]. Neuropathological markers of vascular injury not only often coexist with AD-related amyloid burden, but also seems to correlate well with the degree of this burden. In fact, amyloid burden may conceivably be promoted by microvascular injury. A chronic oligaemic brain condition may predispose to the activation of the amyloidogenic pathway, increasing A-beta oligomerization and deposition [37]. Besides, chronic brain hypoperfusion also seems to stimulate *tau* hyperphosphorylation. BBB dysfunction may affect *β*-amyloid transport between the brain and the periphery, thereby contributing to parenchymal and neurovascular amyloid deposition [37]. Conversely, amyloid may cause vascular injury, as when amyloid-induced inflammatory damage to the endothelium (amyloid angiopathy) [26, 38].

A prospective population-based study with 6,370 elderly subjects found that the risk for both overall dementia and Alzheimer's disease was increased by diabetes [39], with an even higher risk among people treated with insulin (of presumably more severe disease). Other authors have unequivocally confirmed this association in different populations [40–44]. Several studies have demonstrated increased risk of cognitive decline among subjects with Met.S and/or prediabetes [27, 45–48]. Met.S is defined (depending on the clinical criteria utilized) as a constellation of cardiovascular risk factors such as visceral obesity, dyslipidemia, hypertension, and hyperglycemia [49]. Prediabetes is defined as a syndrome presenting with impaired fasting glucose and/or impaired glucose tolerance in which the risk for developing type-2 DM is increased. All the three syndromes above described have insulin resistance and consequent hyperinsulinism as their central pathophysiological feature. The mechanisms by which Met.S components may lead to cognitive decline are depicted in Figure 1. 

Hyperglycemia is a major feature of diabetes [50]. In rodents it is associated with impairment in several cognitive domains. Modifications in brain tissue structure are ultimately attributed to increased activation of polyol and hexosamine pathways, disruption of intracellular second messenger mechanisms, disequilibrium between the generation and scavenging of ROS, and to the accumulation of AGEs, causing important functional and structural modifications in cell proteins [51].

High fasting plasma insulin concentration is a condition that precedes hyperglycemia by many years, predicting DM.2 [52]. This situation seems to be caused by peripheral insulin resistance, which is associated with increased risk of cognitive decline and dementia [45, 53]. Insulin resistance is a condition where the hormone has difficulty in exerting its role, due to receptor dysfunction or alterations in second messenger transduction [28]. Some evidences point towards a positive relationship between peripheral hyperinsulinism and cognitive decline, even in the absence of diabetes. In two studies, lower Mini-Mental State Examination (MMSE) scores were associated with higher insulin serum levels, even when controlled for cardiovascular disease and after excluding diabetic subjects [53, 54]. Possible mechanisms in explaining the role of hyperinsulinism in neurodegeneration are (1) sensibilization of neurons to toxins and other insults in the presence of high insulin concentration levels; (2) decrease transportation of insulin into the brain; (3) *tau* hyperphosphorylation caused by brain insulin resistance; (4) increase in *β*-Amyloid secretion and decrease in its clearance due to competition between Insulin and *β*-Amyloid for the Insulin-Degrading Enzyme (IDE); (5) brain localized hypoglycemic states caused by insulin resistance. 

It was demonstrated that insulin administration to elderly subjects elevates cerebrospinal *β*-Amyloid at 120 min, and this phenomena was correlated with poor cognitive performance [55]. Despite that, it is not yet clear if chronic high serum insulin levels are associated with increased cerebrospinal insulin concentrations. In fact, there are evidences pointing to the contrary [56, 57]. Cerebrospinal fluid (CSF) of people with Alzheimer's disease has lower concentrations of insulin, as compared with controls [58]. This suggests that its relative deficiency, instead of its excess, could be in part responsible for neurodegeneration. 

Acutely, high peripheral insulin levels are associated with increased concentrations of insulin in cerebrospinal fluid [55]. With the chronicity of the hyperinsulinemia, there is a downregulation in transportation mechanisms, which may explain why Alzheimer's patients have lower concentrations of insulin in the cerebrospinal fluid [56, 58]. Furthermore, it is possible that peripheral insulin resistance could decrease insulin transportation into the brain. Brain capillaries of insulin-resistant obese rats have reduced insulin affinity and decreased insulin levels in the hypothalamus [20, 59]. 

This apparent insulin paradox was consistently demonstrated in many other studies with different methodological approaches. At instance, acute administration of intravenous insulin in humans and intraventricular insulin in rats improved memory functions [60, 61]. Recently, it was described that intranasal insulin could properly reach the central nervous system, enhancing memory task performance in humans [62]. In addition, rats trained in tasks involving spatial memory showed increased insulin receptor expression and function [63]. In accordance with previous knowledge that insulin has neurotrophic properties, these data provide evidence that high insulin concentrations improves cognition, at least in acute scenarios. The most reasonable explanation for this apparent paradoxical effect might be attributed to the differences between acute and chronic responses to insulin. Acutely, increases in brain insulin levels have neurotrophic effects, while high chronic insulin levels may have a deleterious effect. In fact, there is evidence for an autoregulatory homeostatic brain mechanism which tries to decrease this state of perpetuated high brain insulin concentration. 

## 5. Insulin Neurotrophic versus Neurotoxic Effects

Insulin might have an effect in sensitization of neurons to excitoxicity. In a rat neuron culture model, it was demonstrated that insulin increases lethal cytotoxic effects of excitatory amino acids, such as glutamate. This effect was not observed with other growth factors and seems to be specific for insulin. Moreover, the effect was retained with different types of excitatory amino acids, suggesting that this phenomenon occur at the intracellular level, rather than in membrane receptor level. Although it has never been demonstrated either *in vivo*, these data reasonably suggest that insulin is a potentially toxic molecule when at very high neuronal concentrations [64, 65].

Despite of this possible neurotoxic effect of insulin in animal models, it is well established that this hormone has an important role as a neuronal survival factor [66, 67]. In fact, insulin seems to protect against the toxic effects of AMPA, oxygen/glucose deprivation and to prevent apoptosis [68–72]. Patients with insulin resistance have loss of insulin signal transduction. Losing the protective effects of insulin, they may be at increased risk for neurodegeneration. 

## 6. Insulin and Alzheimer's Disease

Several cognitive functions seems to be affected by type-2 diabetes mellitus (Table 1). Obese and diabetic people, as well as those with Met.S, are at increased risk of AD [39, 45]. An MRI study compared brains of demented patients with and without diabetes, showing that DM.2 increase cortical atrophy [73]. Older diabetic subjects had increased atrophy of hippocampus, as compared with their paired controls [74]. Thought neither sensible nor specific enough, hippocampal atrophy is one of the earliest neuroradiological signs of AD. Moreover, diffuse cortical atrophy is also more severe among diabetic than nondiabetic Alzheimer's patients [29, 73].

### 6.1. Insulin and *Tau* Phosphorylation

Microtubule Associated Protein (MAP) *Tau* is the molecule responsible for the stabilization of microtubules inside the axons. It determines an efficient and adequate axoplasmic flow, maintaining neuronal connections and turning signal transmission possible [77–79]. One of the hallmarks of AD is the accumulation of aggregates of hyperphosphorylated *tau* protein inside the cells, named neurofibrillary tangles [80]. The exact role for this aggregation in the pathogenesis of AD remains unknown, but it is believed that it could be attributed to an intrinsic neurotoxicity state and/or a deficiency in axoplasmic transport. Others believe that this aggregate is only a reactive protective response against toxic insults. Interestingly, *β*-amiloid toxicity was not observed in the neurons of *tau* knockout mice, suggesting that the formation of neurofibrillary tangles is an essential step for neurodegeneration to occur [81].

It has been demonstrated, in both cell culture and *in vivo *models, that insulin has a pivotal role in regulating *tau* function [82–85]. *Tau* could be phosphorylated at Ser202 residue by extracellular regulated kinase (Erk) family and at Thr231 by glycogen synthase kinase 3 (GSK3). It has been demonstrated in animal models that the rising levels of insulin lead to an activation of Erk, resulting in *tau* phosphorylation at Ser202, and in an increased number of neurofibrillary tangles [86]. Since there is already a strong body of evidence supporting the relationship between hyperinsulinism and cognitive decline, hyperphosphorylation of *tau* could be a plausible implied mechanism for this relationship.

*Tau* is a substrate for caspase activity, and the products of this reaction form molecules that could be phosphorylated by GSK3 [87]. In turn, GSK3 is a constitutively active, proline-directed serine/threonine kinase that plays important roles in a great variety of physiological processes. The decreased activation of insulin receptor caused by insulin resistance results in a decreased phosphorylation of GSK3, turning it more active. The activated GSK3 phosphorylates *tau* and *tau*-derived products at the Thr231 residue, which ultimately accelerates the formation of neurofibrillary tangles [88]. 

The relationship between insulin resistance, hyperinsulinism, and *tau *hyperphosphorylation in the formation of neurofibrillary tangles is, therefore, supported by several lines of evidence [89, 90]. 

### 6.2. Insulin and the *β*-Amyloid Cascade

Deposition of *β*-amyloid plaques in the extracellular space is the other hallmark in the pathology of Alzheimer's disease. These plaques are formed by aggregation of a great variety of misfolded proteins [91]. The most important of them are the Amyloid-*β* (A-beta) peptides 1–40 and 1–42, derived from the *β*-amyloid precursor protein (APP). APP is digested by the proteolytic enzymes beta-secretase and gama-secretase, which produces Abeta40 and Abeta42 peptides. These molecules are prone to form oligomers and to aggregate, producing neurotoxic effects upon neurons. The alternatively route of degradation (nonamyloidogenic pathway) occurs via the *α*-secretase, which cleaves within A-beta region and releases soluble fragments including APP-*α*, a neurotrophic factor par excelance [92]. 

Metabolism of APP is regulated by a great variety of mechanisms, including the influence of certain growth factors, like insulin. In a cell culture model, it was demonstrated that insulin decreased intracellular and increase extracellular concentrations of both A-beta40 and A-beta42 by stimulating intracellular trafficking [93]. Others, using different cell culture models, have showed that insulin deviates the APP metabolism towards the nonamyloidogenic, *α*-APP, pathway. Conversely, with the increase on insulin resistance and its decreased brain actions, occurs as an accumulation of A-beta inside the cell, which could be one of the first mechanisms triggering neurodegeneration in Alzheimer's disease [94]. 

Insulin-degrading enzyme (IDE) is an ubiquitously enzyme, which is highest expressed in liver, testes, muscle, and brain. At the cellular level, it localizes primarily in the cytoplasm and peroxisomes. IDE is another potential explanatory link between insulin and Alzheimer's disease. IDE degrades insulin following its cell internalization, preventing the accumulation of excessive intracellular insulin levels. Beside insulin, IDE binds to a variety of other small peptides, like insulin-like growth factors 1 and 2 (IGF 1 and 2), amylin, and A-beta. These substrates have no homology in their primary structure, although they share similar secondary structures and an amyloidogenic characteristic [95, 96]. 

The interest in the relationship between DM and AD goes beyond a possible cause-effect of the first upon the second. In fact, abnormal amyloid metabolism seems to be a central features in the pathophysiologies of both DM.2 and AD. Amylin deposits in pancreatic islets cells are thought to have a central feature in the pancreatic loss of Langerhans cells associated with DM [97]. Therefore, IDE act as a general regulator of amyloid burden in both the pancreas and the brain. Mutations in the IDE gene and environmental influences which alter its expression might bring a common increased risk to develop both DM.2 and AD [96]. In the particular case of *β*-amyloid plaque formation, IDE is the most important A-beta scavenger protease, being liberated in extracellular space and promoting A-beta cleavage [98]. 

The reaction catalyzed by IDE has different Km depending on the substrate involved. Therefore, subtract competition for IDE degradation should be the rule. Insulin has a lower Km (*∼*0,1 mcM) when compared to A-beta (>2 mcM), what makes this molecule to be faster and preferentially depredated by IDE. In fact, cells culture studies have found that increased insulin concentration competes for IDE degradation with A-beta, slowing its depuration rate [99]. IDE knockout animal models show accumulation of A-beta peptides in the brain [95, 100], while APP mutant mice have reduced plaque formation by increased expression of IDE [101]. Therefore, in states of possible chronic brain high insulin concentrations, A-beta can accumulate, oligomerize, and form plaques, leading to a neurotoxicity and Alzheimer's disease.

Conversely, IDE can be inactivated by increased oxidative stress, as it is often the case in obesity, and, specially, metabolic syndrome [102, 103]. Brains of patients with both Alzheimer's disease and MCI have reduced expression of IDE [104–106]. Furthermore, an allelic IDE gene variant seems to correlate with both A-beta deposition and its plasmatic levels [107, 108]. Therefore, IDE modulatory effect in the etiopathogeny of Alzheimer's disease is already well established. If IDE dysfunction and plaque formation are a cause or a consequence of the rate of brain's oxidative stress, aging, and neurotoxicity is a question waiting for answer. 

## 7. Insulin and Other Growth Factors

In neuron-specific insulin receptor knockout mice (NIRKO), ultrastructural brain morphology and cognitive functions seem to be absolutely normal, since they are not prone to develop any kind of neurodegeneration. In fact, despite the markedly reduced phosphorylation of Akt and GSK3-beta, and the increased phosphorylation of *tau*, NIRKO mice seems to pass through normal development and aging processes [109]. This suggests that insulin resistance is not a determinant of neurodegeneration, but rather a factor that modifies its risk and the rate of its process. 

Insulin partakes the same signaling pathways with other important general growth factors (IGF-1, BDNF, NGF, NT-3, and GDNF) [29]. Taking into account the above data on NIRKO mice, it is reasonable to consider the possibility of a more general growth factor resistance associated with insulin resistance. According to this hypothesis, not only the loss of the proper insulin activity can lead to neurodegeneration, but also disturbances on other important neurotrophic factors, such as the above-mentioned ones. Absence of a well-functioning web of neuroprotective mechanisms may turn the activation of protective signals in response to stressors inefficient, increasing risk of neurodegeneration.

## 8. Conclusions

Almost all forms of neurodegenerative disorders are, early or lately, associated with cognitive decline. Aging-related cognitive dysfunction is a complex event which depends on the net effect of the interactions between many different mechanisms. Even though possibly not acting as primary causative factors, insulin regulation and consequent glucose homeostatic control are important concepts in providing a unified hypothesis by which many age-related neurodegenerative diseases may be related. In fact, AD, PD, and PolyQ disorders share in common the fact that all of them are related to protein misfolding, aggregation, and neurotoxicity. Regardless of the specific genetic mechanisms involved, environmental factors possibly play significant roles in fostering the precise neurodegenerative processes involved in these disorders. Moreover, a common pathway that regulates the accumulation of misfolded proteins at a cellular level, helping the internal cellular machinery to deal with these neurotoxic aggregates could be a very plausible common neuroprotective mechanism against these diseases. 

Dysregulation in the glucose/insulin homeostatic control is an extremely common condition in the modern word and has long been related only with vascular diseases. Recently, the association between insulin resistance and cognitive decline/dementing processes such as AD have become evident. In fact, a great number of researchers worldwide are currently trying to clarify the exact roles of insulin regulation and glycemic control upon the causative processes of AD and other age-related neurodegenerative disease. Knowledge of the specific mechanisms relating insulin dysregulation to neurodegenerative diseases would probably allow the development of drugs that may decelerate neurodegeneration. Meanwhile, improvement of insulin sensitivity and reduction of peripheral hyperinsulinaemia through a healthy diet, weight loss, and aerobic exercise may have beneficial effects in preventing or delaying the progression of AD and other neurodegenerative disorders.

## Figures and Tables

**Table 1:** Summary of cognitive functions found to be affected in Type 2 Diabetes Mellitus (T2DM).

Cognitive functions	T2DM
Verbal memory	↓*
Nonverbal memory	↓*
Attention	↓
Visuospatial performance	—
Processing speed	↓*
Executive function	↓*
Psychomotor efficiency	—
General intelligence	—

Adapted from reference [27], with permission. ↓: decreased;—does not seem to be affected or evidence lacking. Note: domains marked by asterisks have particularly strong supporting data (see [75, 76]).

## References

[1] Timiras PS (2002). *Physiological Basis of Aging and Geriatrics*.

[2] Tschanz JT, Corcoran C, Skoog I (2004). Dementia: the leading predictor of death in a defined elderly population: the Cache County Study. *Neurology*.

[3] Franceschi C, Valensin S, Bonafè M (2000). The network and the remodeling theories of aging: historical background and new perspectives. *Experimental Gerontology*.

[4] Dillin A, Hsu AL, Arantes-Oliveira N (2002). Rates of behavior and aging specified by mitochondrial function during development. *Science*.

[5] Lee SS, Lee RYN, Fraser AG, Kamath RS, Ahringer J, Ruvkun G (2003). A systematic RNAi screen identifies a critical role for mitochondria in C. elegans longevity. *Nature Genetics*.

[6] Bishop NA, Guarente L (2007). Genetic links between diet and lifespan: shared mechanisms from yeast to humans. *Nature Reviews Genetics*.

[7] Mair W, Dillin A (2008). Aging and survival: the genetics of life span extension by dietary restriction. *Annual Review of Biochemistry*.

[8] Finkel T, Holbrook NJ (2000). Oxidants, oxidative stress and the biology of ageing. *Nature*.

[9] Ramasamy R, Vannucci SJ, Yan SSD, Herold K, Yan SF, Schmidt AM (2005). Advanced glycation end products and RAGE: a common thread in aging, diabetes, neurodegeneration, and inflammation. *Glycobiology*.

[10] Takeuchi M, Kikuchi S, Sasaki N (2004). Involvement of advanced glycation end-products (AGEs) in Alzheimer’s disease. *Current Alzheimer Research*.

[11] Guyton AC, Hall JE (2000). *Textbook of Medical Physiology*.

[12] Gerozissis K (2008). Brain insulin, energy and glucose homeostasis; genes, environment and metabolic pathologies. *European Journal of Pharmacology*.

[13] Abbott MA, Wells DG, Fallon JR (1999). The insulin receptor tyrosine kinase substrate p58/53 and the insulin receptor are components of CNS synapses. *Journal of Neuroscience*.

[14] Ferrannini E, Galvan AQ, Gastaldelli A (1999). Insulin: new roles for an ancient hormone. *European Journal of Clinical Investigation*.

[15] Schulingkamp RJ, Pagano TC, Hung D, Raffa RB (2000). Insulin receptors and insulin action in the brain: review and clinical implications. *Neuroscience and Biobehavioral Reviews*.

[16] Niswender KD, Morrison CD, Clegg DJ (2003). Insulin activation of phosphatidylinositol 3-kinase in the hypothalamic arcuate nucleus: a key mediator of insulin-induced anorexia. *Diabetes*.

[17] Sahu A (2003). Leptin signaling in the hypothalamus: emphasis on energy homeostasis and leptin resistance. *Frontiers in Neuroendocrinology*.

[18] Wynne K, Stanley S, McGowan B, Bloom SR (2005). Appetite control. *Journal of Endocrinology*.

[19] Rulifson EJ, Kim SK, Nusse R (2002). Ablation of insulin-producing neurons in files: growth and diabetic phenotypes. *Science*.

[20] Woods SC, Seeley RJ, Baskin DG, Schwartz MW (2003). Insulin and the blood-brain barrier. *Current Pharmaceutical Design*.

[21] Baura GD, Foster DM, Porte D (1993). Saturable transport of insulin from plasma into the central nervous system of dogs in vivo. A mechanism for regulated insulin delivery to the brain. *Journal of Clinical Investigation*.

[22] Wallum BJ, Taborsky GJ, Porte D (1987). Cerebrospinal fluid insulin levels increase during intravenous insulin infusions in man. *Journal of Clinical Endocrinology and Metabolism*.

[23] Kaiyala KJ, Prigeon RL, Kahn SE, Woods SC, Schwartz MW (2000). Obesity induced by a high-fat diet is associated with reduced brain insulin transport in dogs. *Diabetes*.

[24] Wozniak M, Rydzewski B, Baker SP, Raizada MK (1993). The cellular and physiological actions of insulin in the central nervous system. *Neurochemistry International*.

[25] Withers DJ, White M (2000). Perspective: the insulin signaling system—a common link in the pathogenesis of type 2 diabetes. *Endocrinology*.

[26] Craft S (2009). The role of metabolic disorders in Alzheimer disease and vascular dementia: two roads converged. *Archives of Neurology*.

[27] Roriz-Filho S, Sá-Roriz TM, Rosset I (2009). (Pre)diabetes, brain aging, and cognition. *Biochimica et Biophysica Acta*.

[28] Giugliano D, Ceriello A, Esposito K (2008). Glucose metabolism and hyperglycemia. *American Journal of Clinical Nutrition*.

[29] Cole AR, Astell A, Green C, Sutherland C (2007). Molecular connexions between dementia and diabetes. *Neuroscience and Biobehavioral Reviews*.

[30] Wild S, Roglic G, Green A, Sicree R, King H (2004). Global prevalence of diabetes: estimates for the year 2000 and projections for 2030. *Diabetes Care*.

[31] Zhao Y, Ye W, Boye KS, Holcombe JH, Hall JA, Swindle R (2010). Prevalence of other diabetes-associated complications and comorbidities and its impact on health care charges among patients with diabetic neuropathy. *Journal of Diabetes and Its Complications*.

[32] Jarrett RJ (1989). Epidemiology and public health aspects of non-insulin-dependent diabetes mellitus. *Epidemiologic Reviews*.

[33] Stegmayr B, Asplund K (1995). Diabetes as a risk factor for stroke. A population perspective. *Diabetologia*.

[34] Skoog I (1994). Risk factors for vascular dementia: a review. *Dementia*.

[35] Viswanathan A, Rocca WA, Tzourio C (2009). Vascular risk factors and dementia: how to move forward?. *Neurology*.

[36] Jellinger KA (2008). The pathology of “vascular dementia”: a critical update. *Journal of Alzheimer’s Disease*.

[37] Pluta R, Ułamek M (2008). Brain ischemia and ischemic blood-brain barier as etiological factors in sporadic Alzheimer’s disease. *Neuropsychiatric Disease and Treatment*.

[38] Air EL, Kissela BM (2007). Diabetes, the metabolic syndrome, and ischemic stroke: epidemiology and possible mechanisms. *Diabetes Care*.

[39] Ott A, Stolk RP, van Harskamp F, Pols HAP, Hofman A, Breteler MMB (1999). Diabetes mellitus and the risk of dementia: the Rotterdam Study. *Neurology*.

[40] Biessels GJ, Deary IJ, Ryan CM (2008). Cognition and diabetes: a lifespan perspective. *The Lancet Neurology*.

[41] Luchsinger JA, Tang MX, Stern Y, Shea S, Mayeux R (2001). Diabetes mellitus and risk of Alzheimer’s disease and dementia with stroke in a multiethnic cohort. *American Journal of Epidemiology*.

[42] Peila R, Rodriguez BL, Launer LJ (2002). Type 2 diabetes, APOE gene, and the risk for dementia and related pathologies: the Honolulu-Asia Aging Study. *Diabetes*.

[43] MacKnight C, Rockwood K, Awalt E, McDowell I (2002). Diabetes mellitus and the risk of dementia, Alzheimer’s disease and vascular cognitive impairment in the Canadian Study of Health and Aging. *Dementia and Geriatric Cognitive Disorders*.

[44] Arvanitakis Z, Wilson RS, Bienias JL, Evans DA, Bennett DA (2004). Diabetes mellitus and risk of Alzheimer disease and decline in cognitive function. *Archives of Neurology*.

[45] Luchsinger JA, Tang MX, Shea S, Mayeux R (2004). Hyperinsulinemia and risk of Alzheimer disease. *Neurology*.

[46] Yaffe K, Blackwell T, Kanaya AM, Davidowitz N, Barrett-Connor E, Krueger K (2004). Diabetes, impaired fasting glucose, and development of cognitive impairment in older women. *Neurology*.

[47] Roriz-Cruz M, Rosset I, Wada T (2007). Cognitive impairment and frontal-subcortical geriatric syndrome are associated with metabolic syndrome in a stroke-free population. *Neurobiology of Aging*.

[48] van den Berg E, Dekker JM, Nijpels G (2008). Cognitive functioning in elderly persons with type 2 diabetes and metabolic syndrome: the Hoorn study. *Dementia and Geriatric Cognitive Disorders*.

[49] Matsuo Y, Hashizume T, Shioji S, Akasaka T (2008). Metabolic syndrome is strongly associated with chronic subclinical inflammation in patients achieving optimal low-density lipoprotein-cholesterol levels in secondary prevention of cardiovascular disease. *Circulation Journal*.

[50] Gispen WH, Biessels GJ (2000). Cognition and synaptic plasticity in diabetes mellitus. *Trends in Neurosciences*.

[51] Brownlee M (2001). Biochemistry and molecular cell biology of diabetic complications. *Nature*.

[52] Weyer C, Hanson RL, Tataranni PA, Bogardus C, Pratley RE (2000). A high fasting plasma insulin concentration predicts type 2 diabetes independent of insulin resistance: evidence for a pathogenic role of relative hyperinsulinemia. *Diabetes*.

[53] Kuusisto J, Koivisto K, Mykkänen L (1997). Association between features of the insulin resistance syndrome and Alzheimer’s disease independently of apolipoprotein E4 phenotype: cross sectional population based study. *British Medical Journal*.

[54] Stolk RP, Breteler MMB, Ott A (1997). Insulin and cognitive function in an elderly population the rotterdam study. *Diabetes Care*.

[55] Watson GS, Peskind ER, Asthana S (2003). Insulin increases CSF A*β*42 levels in normal older adults. *Neurology*.

[56] Craft S, Peskind E, Schwartz MW, Schellenberg GD, Raskind M, Porte D (1998). Cerebrospinal fluid and plasma insulin levels in Alzheimer’s disease: relationship to severity of dementia and apolipoprotein E genotype. *Neurology*.

[57] Molina JA, Jiménez-Jiménez FJ, Vargas C (2002). Cerebrospinal fluid levels of insulin in patients with Alzheimer’s disease. *Acta Neurologica Scandinavica*.

[58] Steen E, Terry BM, Rivera EJ (2005). Impaired insulin and insulin-like growth factor expression and signaling mechanisms in Alzheimer’s disease—is this type 3 diabetes?. *Journal of Alzheimer’s Disease*.

[59] Schwartz MW, Figlewicz DF, Kahn SE, Baskin DG, Greenwood MRC, Porte D (1990). Insulin binding to brain capillaries is reduced in genetically obese, hyperinsulinemic Zucker rats. *Peptides*.

[60] Craft S, Asthana S, Newcomer JW (1999). Enhancement of memory in Alzheimer disease with insulin and somatostatin, but not glucose. *Archives of General Psychiatry*.

[61] Park CR, Seeley RJ, Craft S, Woods SC (2000). Intracerebroventricular insulin enhances memory in a passive-avoidance task. *Physiology and Behavior*.

[62] Reger MA, Watson GS, Green PS (2008). Intranasal insulin improves cognition and modulates *β*-amyloid in early AD. *Neurology*.

[63] Zhao W, Chen H, Xu H (1999). Brain insulin receptors and spatial memory. Correlated changes in gene expression, tyrosine phosphorylation, and signaling molecules in the hippocampus of water maze trained rats. *Journal of Biological Chemistry*.

[64] Schäfer M, Erdö SL (1991). Development of glutamate neurotoxicity in cortical cultures: induction of vulnerability by insulin. *Developmental Brain Research*.

[65] Schäfer M, Erdö SL (1992). Insulin-specific sensitization of cultured cerebrocortical neurons to glutamate excitotoxicity. *Brain Research*.

[66] Díaz B, Serna J, de Pablo F, de la Rosa EJ (2000). In vivo regulation of cell death by embryonic (pro)insulin and the insulin receptor during early retinal neurogenesis. *Development*.

[67] Li ZG, Zhang W, Sima AAF (2005). The role of impaired insulin/IGF action in primary diabetic encephalopathy. *Brain Research*.

[68] Kim SJ, Han Y (2005). Insulin inhibits AMPA-induced neuronal damage via stimulation of protein kinase B (Akt). *Journal of Neural Transmission*.

[69] Mielke JG, Yu TW (2005). Insulin exerts neuroprotection by counteracting the decrease in cell-surface GABA receptors following oxygen-glucose deprivation in cultured cortical neurons. *Journal of Neurochemistry*.

[70] Ryu BR, Ko HW, Jou I, Noh JS, Gwag BJ (1999). Phosphatidylinositol 3-kinase-mediated regulation of neuronal apoptosis and necrosis by insulin and IGF-I. *Journal of Neurobiology*.

[71] Nakamura M, Barber AJ, Antonetti DA (2001). Excessive hexosamines block the neuroprotective effect of insulin and induce apoptosis in retinal neurons. *Journal of Biological Chemistry*.

[72] Tanaka M, Sawada M, Yoshida S, Hanaoka F, Marunouchi T (1995). Insulin prevents apoptosis of external granular layer neurons in rat cerebellar slice cultures. *Neuroscience Letters*.

[73] Biessels GJ, De Leeuw FE, Lindeboom J, Barkhof F, Scheltens P (2006). Increased cortical atrophy in patients with Alzheimer’s disease and type 2 diabetes mellitus. *Journal of Neurology, Neurosurgery and Psychiatry*.

[74] den Heijer T, Vermeer SE, van Dijk EJ (2003). Type 2 diabetes and atrophy of medial temporal lobe structures on brain MRI. *Diabetologia*.

[75] Kodl CT, Seaquist ER (2008). Cognitive dysfunction and diabetes mellitus. *Endocrine Reviews*.

[76] Awad N, Gagnon M, Messier C (2004). The relationship between impaired glucose tolerance, type 2 diabetes, and cognitive function. *Journal of Clinical and Experimental Neuropsychology*.

[77] Cleveland DW, Hwo SY, Kirschner MW (1977). Purification of *tau*, a microtubule associated protein that induces assembly of microtubules from purified tubulin. *Journal of Molecular Biology*.

[78] Drubin DG, Kirschner MW (1986). Tau protein function in living cells. *Journal of Cell Biology*.

[79] Weingarten MD, Lockwood AH, Hwo SY, Kirschner MW (1975). A protein factor essential for microtubule assembly. *Proceedings of the National Academy of Sciences of the United States of America*.

[80] Trojanowski JQ, Lee VMY (2002). The role of *tau* in Alzheimer’s disease. *Medical Clinics of North America*.

[81] Rapoport M, Dawson HN, Binder LI, Vitek MP, Ferreira A (2002). Tau is essential to *β*-amyloid-induced neurotoxicity. *Proceedings of the National Academy of Sciences of the United States of America*.

[82] Hong M, Lee VMY (1997). Insulin and insulin-like growth factor-1 regulate *tau* phosphorylation in cultured human neurons. *Journal of Biological Chemistry*.

[83] Lesort M, Jope RS, Johnson GVW (1999). Insulin transiently increases *tau* phosphorylation: involvement of glycogen synthase kinase-3*β* and Fyn tyrosine kinase. *Journal of Neurochemistry*.

[84] Schubert M, Brazil DP, Burks DJ (2003). Insulin receptor substrate-2 deficiency impairs brain growth and promotes *tau* phosphorylation. *Journal of Neuroscience*.

[85] Lesort M, Johnson GVW (2000). Insulin-like growth factor-1 and insulin mediate transient site-selective increases in *tau* phosphorylation in primary cortical neurons. *Neuroscience*.

[86] Freude S, Plum L, Schnitker J (2005). Peripheral hyperinsulinemia promotes *tau* phosphorylation in vivo. *Diabetes*.

[87] Cho JH, Johnson GVW (2004). Glycogen synthase kinase 3*β* induces caspase-cleaved *tau* aggregation in situ. *Journal of Biological Chemistry*.

[88] Cho JH, Johnson GVW (2003). Glycogen synthase kinase 3*β* phosphorylates *tau* at both primed and unprimed sites: differential impact on microtubule binding. *Journal of Biological Chemistry*.

[89] Gasparini L, Xu H (2003). Potential roles of insulin and IGF-1 in Alzheimer’s disease. *Trends in Neurosciences*.

[90] Craft S, Watson GS (2004). Insulin and neurodegenerative disease: shared and specific mechanisms. *The Lancet Neurology*.

[91] Kovacech B, Zilka N, Novak M (2009). New age of neuroproteomics in Alzheimer’s disease research. *Cellular and Molecular Neurobiology*.

[92] Selkoe DJ (1998). The cell biology *β*-amyloid precursor protein and presenilin in Alzheimer’s disease. *Trends in Cell Biology*.

[93] Gasparini L, Gouras GK, Wang R (2001). Stimulation of *β*-amyloid precursor protein trafficking by insulin reduces intraneuronal *β*-amyloid and requires mitogen-activated protein kinase signaling. *Journal of Neuroscience*.

[94] Solano DC, Sironi M, Bonfini C, Solerte SB, Govoni S, Racchi M (2000). Insulin regulates soluble amyloid precursor protein release via phosphatidyl inositol 3 kinase-dependent pathway. *FASEB Journal*.

[95] Farris W, Mansourian S, Chang Y (2003). Insulin-degrading enzyme regulates the levels of insulin, amyloid *β*-protein, and the *β*-amyloid precursor protein intracellular domain in vivo. *Proceedings of the National Academy of Sciences of the United States of America*.

[96] Qiu WQ, Folstein MF (2006). Insulin, insulin-degrading enzyme and amyloid-*β* peptide in Alzheimer’s disease: review and hypothesis. *Neurobiology of Aging*.

[97] Kahn SE, Andrikopoulos S, Verchere CB (1999). Islet amyloid: a long-recognized but underappreciated pathological feature of type 2 diabetes. *Diabetes*.

[98] Ling Y, Morgan K, Kalsheker N (2003). Amyloid precursor protein (APP) and the biology of proteolytic processing: relevance to Alzheimer’s disease. *International Journal of Biochemistry and Cell Biology*.

[99] Qiu WQ, Ye Z, Kholodenko D, Seubert P, Selkoe DJ (1997). Degradation of amyloid *β*-protein by a metalloprotease secreted by microglia and other neural and non-neural cells. *Journal of Biological Chemistry*.

[100] Miller BC, Eckman EA, Sambamurti K (2003). Amyloid-*β* peptide levels in brain are inversely correlated with insulysin activity levels in vivo. *Proceedings of the National Academy of Sciences of the United States of America*.

[101] Leissring MA, Farris W, Chang AY (2003). Enhanced proteolysis of *β*-amyloid in APP transgenic mice prevents plaque formation, secondary pathology, and premature death. *Neuron*.

[102] Caccamo A, Oddo S, Sugarman MC, Akbari Y, LaFerla FM (2005). Age- and region-dependent alterations in A*β*-degrading enzymes: implications for A*β*-induced disorders. *Neurobiology of Aging*.

[103] Shinall H, Song ES, Hersh LB (2005). Susceptibility of amyloid *β* peptide degrading enzymes to oxidative damage: a potential Alzheimer’s disease spiral. *Biochemistry*.

[104] Zhao Z, Xiang Z, Haroutunian V, Buxbaum JD, Stetka B, Pasinetti GM (2007). Insulin degrading enzyme activity selectively decreases in the hippocampal formation of cases at high risk to develop Alzheimer’s disease. *Neurobiology of Aging*.

[105] Pérez A, Morelli L, Cresto JC, Castaño EM (2000). Degradation of soluble amyloid *β*-peptides 1-40, 1-42, and the Dutch variant 1-40Q by insulin degrading enzyme from Alzheimer disease and control brains. *Neurochemical Research*.

[106] Cook DG, Leverenz JB, McMillan PJ (2003). Reduced hippocampal insulin-degrading enzyme in late-onset Alzheimer’s disease is associated with the apolipoprotein E-*ε*4 allele. *American Journal of Pathology*.

[107] Björk BF, Katzov H, Kehoe P (2007). Positive association between risk for late-onset Alzheimer disease and genetic variation in IDE. *Neurobiology of Aging*.

[108] Ertekin-Taner N, Allen M, Fadale D (2004). Genetic variants in a haplotype block spanning IDE are significantly associated with plasma A*β*42 levels and risk for Alzheimer disease. *Human Mutation*.

[109] Schubert M, Gautam D, Surjo D (2004). Role for neuronal insulin resistance in neurodegenerative diseases. *Proceedings of the National Academy of Sciences of the United States of America*.

